# Lysosomal dysfunction in proteinopathic neurodegenerative disorders: possible therapeutic roles of cAMP and zinc

**DOI:** 10.1186/s13041-019-0439-2

**Published:** 2019-03-12

**Authors:** Jae-Young Koh, Ha Na Kim, Jung Jin Hwang, Yang-Hee Kim, Sang Eun Park

**Affiliations:** 1 Department of Neurology, University of Ulsan College of Medicine, Seoul, South Korea; 2 Neural Injury Lab, Biomedical Research Center, Asan Institute for Life Sciences, Asan Medical Center, Seoul, South Korea; 3 Department of Convergence Medicine, University of Ulsan College of Medicine, Asan Medical Center, Seoul, South Korea; 4 Department of Integrative Bioscience and Biotechnology, Sejong University, Seoul, South Korea; 5 Asan Institute for Life Sciences, Asan Medical Center, Seoul, South Korea

**Keywords:** Lysosome, cAMP, Zinc, MT3, EALP

## Abstract

A number of neurodegenerative diseases, including Alzheimer’s disease, Parkinson’s disease, and amyotrophic lateral sclerosis, share intra- and/or extracellular deposition of protein aggregates as a common core pathology. While the species of accumulating proteins are distinct in each disease, an increasing body of evidence indicates that defects in the protein clearance system play a crucial role in the gradual accumulation of protein aggregates. Among protein degradation systems, the endosome-autophagosome-lysosome pathway (EALP) is the main degradation machinery, especially for large protein aggregates. Lysosomal dysfunction or defects in fusion with vesicles containing cargo are commonly observed abnormalities in proteinopathic neurodegenerative diseases. In this review, we discuss the available evidence for a mechanistic connection between components of the EALP-especially lysosomes-and neurodegenerative diseases. We also focus on lysosomal pH regulation and its significance in maintaining flux through the EALP. Finally, we suggest that raising cAMP and free zinc levels in brain cells may be beneficial in normalizing lysosomal pH and EALP flux.

## Introduction: Contribution of lysosomal dysfunction to the pathogenesis of neurodegenerative disorders

The survival and health of a eukaryotic cell depends on maintenance of a homeostatic state of continuous generation and degradation of cellular macroconstituents, such as membrane lipids, proteins, and organelles. If a cellular degradation process becomes compromised, abnormal proteins, lipids, and dysfunctional organelles accumulate. Accumulation of certain waste proteins can lead to the formation of toxic protein oligomers and aggregates [1]. In addition, remnants of dysfunctional organelles, such as mitochondria and peroxisomes, due to the lysosomal dysfunction may contribute to an excessive generation of reactive oxygen species (ROS) [2]. Taken together, these events lead to severe cellular dysfunction and, ultimately, cell death.

Macromolecular degradation occurs in diverse cellular compartments, including proteasomes, peroxisomes, and lysosomes. Lysosomes are specialized for the all-purpose, high-capacity degradation of large proteins, protein aggregates, and organelles [3]. Cargoes are delivered to lysosomes via diverse routes that include autophagy, endocytosis and phagocytosis, collectively referred to as the endosome-autophagosome-lysosome pathway (EALP). By definition, an endosome is a membrane-bound organelle. It is a component of the endocytic membrane transport pathway originating from the trans-Golgi membrane. Endosomes provide an environment for material to be sorted before it reaches the degradative lysosome. Autophagosome, also called initial autophagic vacuoles (AVi), is a double-membrane bound vesicle, which doesn’t yet contain lysosomal membrane proteins and enzymes. After fusion with lysosome,s autophagosomes are called autolysosomes. Finally, lysosome is the organelle containing various proteolytic enzymes. It functions as the cellular digestive organ [3–5]. As the site of degradation in the EALP, lysosomes contain more than 60 hydrolases that act in concert to degrade almost all cellular macroconstituents [6]. All of these enzymes function optimally at the acidic pH (4.5–5.5) of the lysosomal lumen [6]. Although the mode of activation of each enzyme may differ, autocatalytic conversion of a proform to an active form seems to be the main mechanism for many lysosomal enzymes, such as cathepsins [7].

In humans and other mammals that have a relatively long life span, the maintenance of adequate lysosomal function is especially important for the health of post-mitotic neurons in central nervous system (CNS) that are destined to survive the entire lifetime of the organism. Therefore, lysosomal dysfunction tends to affect the CNS to a greater extent than other tissues or organs in humans. One example of such dysfunctions is a group of disorders termed lysosomal storage disorders (LSDs), which include neuronal ceroid lipofuscinosis (NCL, also known as Batten’s disease), Niemann-Pick type C (NPC), and Mucolipidosis type IV (MLIV). Although these diseases are caused by defects in different genes, specifically *CLN1-CLN3*, *NPC1*, and *MCOLN1* (transient receptor potential mucolipin channel 1, TRPML1), respectively, all of these genetic defects converge on the common consequence of lysosomal dysfunction [8].

Another example is a group of age-related neurodegenerative disorders that includes Alzheimer’s disease (AD), Parkinson’s disease (PD), and amyotrophic lateral sclerosis (ALS). In addition to these neurodegenerative diseases, aging itself is accompanied by lysosomal dysfunction. It has been reported that lysosomal proteolytic activity declines with aging; as a consequence, damaged organelles and mutated toxic proteins accumulate over time [9]. Lysosomal accumulation of lipofuscin, a non-degradable pigmented polymeric substance, serves as an indicator of lysosomal dysfunction. Lipofuscin accumulation further inhibits lysosomal degradative activity [10], fostering gradual accumulation of other age-related waste products in lysosomes. Because aging is the most definitive common risk factor for neurodegenerative disorders such as AD and PD, the age-dependent decline in lysosomal function may lay the groundwork for the accumulation of protein aggregates such as amyloid beta (Aβ), tau, and α-synuclein under the respective neurodegenerative conditions [4]. As noted above, some of these protein aggregates, in turn, may cause further lysosomal dysfunction, creating a vicious cycle that leads to progressive increases in protein aggregation and lysosomal dysfunction. Hence, regardless of how it is triggered, be it by aging or specific genetic defects, lysosomal dysfunction not only provides fertile soil for initiating diverse neurodegenerative conditions, it also contributes to disease progression.

## Factors that govern overall lysosomal functions

The proper maintenance of lysosomal functions requires that several parameters be held within a suitable range.

The first is having a **sufficient quantity/total volume of lysosomes** to meet the demand at a particular moment. Depending on cell type, the number of lysosomes varies between 50 and 1000 per cell [11]. The size of lysosomes is in the range of 0.2–0.8 μm in diameter [12], and their combined volume accounts for about 1–15% of the total cell volume [13]. The demand for degradation in a cell changes dynamically depending on a number of factors, including the rate of protein synthesis, the rate of endocytosis, the amount of organelle damage, and energy requirements (e.g., under starvation conditions) among others. To manage such continuously changing demands for degradation, cells must be equipped with mechanisms to rapidly adjust lysosomal quantity accordingly. At the transcription level, the Microphthalmia family of bHLH-LZ transcription factors (MiT/TFE) play important roles in lysosomal biogenesis. Especially, transcription factor EB (TFEB), one of MiT/TFE family, and ZKSCAN3 (zinc finger with KRAB and SCAN domains 3) function as major activator and inhibitor, respectively, of lysosomal biogenesis [14, 15]. However, following a transcription-based increase, for example through TFEB activation, how the quantity of lysosomes returns to baseline levels is not clearly understood. After the increase, some lysosomes lose their lysosomal membrane markers and/or luminal enzymes, and are recycled to generate other membrane-bound organelles [16]. In addition, some of the lysosomal membrane may be incorporated into the plasma membrane, endoplasmic reticulum (ER), or Golgi [17–19]. The biogenesis of lysosomes is also not fully elucidated. Late endosomes may lose endosomal membrane markers, such as Rab7, and acquire lysosomal enzymes and membrane proteins, such as LAMP1 (lysosomal-associated membrane protein 1) and LAMP2, and in the process transmorph into lysosomes [20]. Lysosomes are also regenerated from autolysosomes through a process termed autophagic lysosome reformation (ALR) in which proto-lysosomes bud from autolysosomes [21]. In this process, clathrin (and its adaptor proteins), actin polymerization, and PI (4,5) P2 play key roles [22]. In addition, inhibition of phosphoinositide 5-kinase (PIKFYVE), an enzyme critical for the synthesis of PI (3,5) P2, results in the failure of lysosome biogenesis and the accumulation of vacuoles [23, 24]. These reports indicate that different phosphoinositol lipids are involved in many steps of lysosomal regeneration.

The second parameter for maintenance of proper lysosomal function is **adequate formation of cargoes and their delivery to lysosomes—packaging, transport, and fusion**. There are two systems for delivering large cargoes to lysosomes in a cell: macroautophagy (via autophagosomes) and endocytosis (via endosomes). Defects in these processes are frequently observed in neurons of neurodegenerative diseases. A reduction in autophagophore formation, the initial event in autophagy, results in accumulation of waste proteins and organelles in the cytosol without accumulation of autophagosomes as vacuoles. For instance, a decrease in beclin-1 expression leads to accumulation of mutant huntingtin protein in Huntington’s disease (HD) and loss of laforin polyglucosan inclusions in Lafora’s body disease via activation of mTOR (mammalian/mechanistic target of rapamycin) signaling pathway [25, 26]. Similarly, defects in endocytosis can result in the accumulation of membrane proteins in the plasma membrane, with little vacuole accumulation. For example, neurodegenerative disease-related protein aggregates, such as polyglutamine, huntingtin, ataxin-1, and superoxide dismutase-1, block clathrin-mediated endocytosis and intracellular trafficking in neurodegenerative disease [27]. In contrast, defects in vesicle transport result in the accumulation of vacuoles derived from endosomes or autophagosomes. In neurons, especially at axon terminals, endocytosed materials require retrograde transport along microtubules in long axons to the cell body, where mature lysosomes normally reside. Defects in this transport process result in the accumulation of endosomes somewhere along the axons [28]. In the case of macroautophagy, a microtubule-based transport system involving interactions with LC3 is also involved [29]. Deficits in this process also contribute to neurodegenerative disease [26]. Finally, defects in fusion between cargo-containing autophagosomes and lysosomes also result in the accumulation of autophagosomes and/or endosomes. A reduction in autolysosome formation can lead to the accumulation of waste proteins and damaged organelles inside double-membrane autophagosomes. Various drugs that alkalinize the lysosomal lumen, for instance chloroquine, have been reported to inhibit fusion of lysosomes and autophagosomes [30]. Presenilin-1 (PSEN1) has recently been shown to play a role in maintaining lysosomal acidity [31, 32]. Hence, it is likely that a deficiency in PSEN1 function results in lysosomal alkalinization and defective fusion. Interestingly, it has been reported that lysosomal pH tends to shift toward a more alkaline direction with aging alone [33], suggesting that fusion of cargo-containing vesicles with lysosome becomes compromised with age.

The third parameter is a **sufficient quantity and adequate quality of lysosomal enzymes**. While the detailed mechanisms responsible for regulating the quantity of lysosomal enzymes have yet to be fully delineated, the transcription factors TFEB and ZKSCAN3 again have been found to play crucial roles as activator and inhibitor, respectively [14, 15]. As master switches, these transcription factors link changing cellular demands to appropriate synthesis of lysosomal enzymes. After synthesis, most lysosomal enzymes leave the trans-Golgi network (TGN) after their modification with mannose-6-phosphate (M6P) residues [34]. These M6P-modified enzymes can then be transported to the endosomal/lysosomal system through interactions with M6P receptors (MPRs). Alternatively, some enzymes are recognized by lysosomal integral membrane protein-2 (LIMP-2) and sortilin, and subsequently transported to lysosomes [35]. Any defect in these processes may cause a deficiency in lysosomal enzymes. In addition to quantity, the quality of lysosomal enzymes is important; mutations in genes encoding lysosomal enzymes result in enzyme deficiency and reduced degradation of particular substrates, causing their accumulation in lysosomes. For instance, Gaucher disease (GD), a lysosomal storage disorder, come from an inherited deficiency of lysosomal glucocerebrosidase (GCase) arising from mutations in the gene *glucosylceramidase (GBA)* [36, 37]. GCase deficiency caused by GBA mutations interferes with the degradation of α-synuclein [38]. Patients with GD show parkinsonian symptoms, meanwhile, GBA mutations are more frequently observed in patients with PD. Thus, the adequate quality of the lysosomal enzyme is also crucial for lysosomal function and is associated with the onset of neurodegenerative diseases.

Finally, **the lysosomal lumen milieu must be adequately controlled for enzymes to function optimally.** One of the most critical known variables is luminal pH. Because most lysosomal enzymes function optimally at an acidic pH, the lysosomal lumen is kept slightly acidic, mainly owing to the action of vacuolar ATPase (V-ATPase), which moves protons (H^+^) from the cytosol to the lysosomal lumen against a concentration gradient using the energy of ATP hydrolysis [39]. Abnormalities in the function of V-ATPase result in an overall decrease in lysosomal degradation [40, 41]. Another potential candidate that may affect lysosomal pH is the Na^+^/H^+^ exchanger (NHE). Although the presence of NHEs in the endosomal membrane and their function in lysosomal biogenesis have been demonstrated [42], their role in regulating lysosomal pH is not yet clear. In addition to H^+^, lysosomal enzymes may require adequate levels of other ions, such as calcium (Ca^2+^), iron (Fe^2+^) and zinc (Zn^2+^). Various cation channels may take part in homeostasis of these ions in the lysosomes including TRPML1–3 (also called mucolipins), two-pore channels (TPCs) [43] and transporters, such as Zn^2+^ transporter 2 (ZnT2), ZnT4 and ATP13A2 (ATPase cation transporting 13A2, also known as PARK9). For instance, mutation of *TRPML1* causes neurodegeneration through the accumulation of lipofuscin in lysosomes [44, 45]. In fact, loss of TRPML function results in dyshomeostases of intracellular Ca^2+^, Fe^2+^, and Zn ^2+^ as well as abnormal lysosomal pH [45, 46]. A loss-of-function mutation in the gene encoding *ATP13A2/PARK9*, a putative Zn^2+^ transporter in intracellular vesicles, decreases autophagy-lysosomal pathway-associated vesicular Zn^2+^, alters expression of Zn^2+^ transporters, and increases sensitivity to Zn^2+^ [47–49]. These events lead to lysosomal dysfunction and accumulation of α-synuclein in PD. These channels and transporters may also take part in signaling lysosomal distress to nuclei. For instance, under starvation conditions, Ca^2+^ released into the cytosol via the TRPML1 channel activates the Ca^2+^-dependent phosphatase calcineurin, leading to dephosphorylation of TFEB and its translocation across the nuclear envelope into the nucleus, where it induces lysosomal biogenesis [50, 51]. Interestingly, starvation-induced nuclear translocation of TFEB is reduced in fibroblasts from patients with MLIV or by knockdown of TRPML1 [50].

## Evidence for lysosomal dysfunction in certain neurodegenerative diseases

If cargo delivery to lysosomes were compromised and/or lysosomal degradation were suboptimal, a variety of waste products would accumulate in cells. Although some waste products can be degraded by proteasomes or get secreted as exosomes through the formation of multivesicular bodies (MVBs), a substantial fraction remains and accumulates in organelles and the cytosol, disrupting various cell functions. It is not yet clear which waste products are particularly neurotoxic, but in neurodegenerative conditions, oligomers of peptides and proteins such as Aβ, phospho-tau, α-synuclein, and TDP-43 (encoded by the *TARDBP* gene) are the main culprits.

Aβ, the main component of amyloid plaques in AD, is produced from the plasma membrane protein amyloid precursor protein (APP), mainly in late endosomes, by the action of beta secretase-1 (BACE1). BACE1 is a transmembrane aspartic protease responsible for most of the β-secretase activity, but there is no direct evidence to support a causative role for increased BACE1 activity in AD. Instead, a growing body of evidence indicates that Aβ is a normal product of APP metabolism that serves diverse physiological functions [52, 53]. For instance, a recent study reported that Aβ acts as an antimicrobial peptide in the brain [54]. Since BACE1 works within a narrow pH range, with peak activity at pH 4.5 [55, 56], prolonged residence of APP in acidic late endosomes, as may occur under conditions of lysosomal dysfunction, could be a contributing factor to the increase in Aβ. Taken together with decreased lysosomal degradation, this late-endosomal retention of BACE1 would culminate in the accumulation of Aβ in late endosomes, cytosol, and possibly in exosomes. In fact, Aβ itself can cause lysosomal alkalinization and dysfunction [57]. Accordingly, a small increase in Aβ production caused by APP mutations could induce mild lysosomal dysfunction, which, in turn, leads to further increases in Aβ levels. This type of a positive feedback loop would result in a gradual increase in Aβ accumulation. Intriguingly in this context, mutant PSEN1, which has been suggested to increase Aβ levels by virtue of its role as a component of γ-secretase, also causes lysosomal alkalinization by inhibiting V-ATPase assembly [31, 58]. Hence, a common denominator in the effects of both mutated APP and PS-1 may be lysosomal dysfunction.

Another potential contributor to aberrant lysosomal degradation in AD is hyperphosphorylation and accumulation of tau, which leads to the formation of neurofibrillary tangles, another hallmark of AD. Normally, tau associates with and stabilizes microtubules. Dissociation of tau from microtubules disrupts retrograde transport of peripherally derived endosomes to perikaryal lysosomes, interfering with degradation of endosomal cargoes. Conversely, inhibition of lysosomal degradation aggravates phospho-tau accumulation [59]. Although mutations in the tau gene cause tauopathies without producing conspicuous Aβ accumulation [60], mutations in APP genes cause accumulation of Aβ, α-synuclein, and tau [61, 62]. These findings indicate that aberrant APP processing and Aβ may have broader effects on the EALP than tau. Alternatively, lysosomal dysfunction alone may not be sufficient for Aβ accumulation.

Recent studies have found that a substantial fraction of genes involved in PD are related to endosomal trafficking and/or lysosomal function, including *VPS35, GBA, ATP13A2, ATP6AP2, DNAJC13/RME-8, RAB7L1*, and GAK (cyclin G-associated kinase) [63]. For instance, the *ATP13A2/PARK9* gene encodes a lysosomal ATPase that transports cations, and the *ATP6AP2* gene encodes a transmembrane protein that is a component of V-ATPase. The resultant functional defects in lysosomes likely contribute to accumulation of α-synuclein aggregates in midbrain dopaminergic neurons as well as cortical neurons. Reciprocally, as in case of Aβ in AD, thus-formed α-synuclein aggregates can further impair macroautophagy [64, 65], again giving rise to a vicious cycle.

In addition to their involvement in AD and PD, lysosomal dysfunction has been implicated in the pathogenesis of other neurodegenerative diseases, including ALS, HD, and other trinucleotide repeat disorders [66–68]. Aggregates of SOD-1 (superoxide dismutase-1) or TDP-43, which are associated with ALS, disrupt the EALP [69–72]. Aggregates of mutant huntingtin, and likely those of other polyglutamine proteins, also inhibit the EALP [67]. Hence, it is tempting to speculate that a common mechanism underlying neurodegenerative diseases, especially those accompanied by accumulation of aggregated proteins, may be lysosomal or EALP dysfunction. Hence, therapies that normalize EALP function may be efficacious in diverse neurodegenerative diseases.

## How to restore lysosomal function

The EALP is a complex pathway regulated by a number of kinases, membrane proteins, transport machinery, signaling membrane phospholipids and cations, such as Ca^2+^, Mg^2+^, Fe^2+^ and Zn^2+^ [73–77]. It is not an easy task to determine which step is most critically affected in a particular neurodegenerative condition. Intensive efforts have been undertaken to find ways to activate autophagy at the autophagosome-formation step. For instance, inhibitors of mTORC1 (mammalian/mechanistic target of rapamycin complex 1), such as rapamycin, activate the ULK (Unc-51–like autophagy activating kinase) complex, which is necessary for autophagosome formation. In theory, however, if the main defect is lysosomal dysfunction causing arrested autophagy, activation of autophagosome formation alone may not be sufficient to restore lysosomal protein degradation. Although it has been reported that levels of beclin-1 are reduced in AD brains [78, 79], no direct evidence for abnormalities in autophagosome formation exists. Instead, a growing body of evidence indicates that autophagosomes, as well as late endosomes, likely accumulate as a result of inefficient fusion between cargo-containing vesicles and lysosomes [80, 81]. Consistent with this, a recent study presented evidence that autophagosome formation is not reduced, but is instead upregulated, in the early stage of AD [82].

On the other hand, as discussed above, there is ample evidence that lysosomal dysfunction plays a role in neurodegenerative disorders. One way to increase lysosomal function is to upregulate lysosomal proteins, including enzymes. Although the level of TFEB, the master transcriptional activator of lysosomal proteins, is not reduced in AD brains, further increasing it by delivering viral TFEB constructs has been shown to reduce Aβ and phospho-tau levels in AD mice [83, 84]. Hence, TFEB may be a viable target for the development of drugs that boost lysosomal degradation. There appear to be diverse ways to increase the level and activity of TFEB in addition to introducing the corresponding gene. For example, AKT, mTORC1, and ERK-2 (extracellular signal-regulated kinase-2) phosphorylate TFEB to inhibit its translocation to nuclei; thus, inhibitors of these kinases may upregulate TFEB activity. Notably, the disaccharide sugar trehalose activates TFEB and induces lysosomal biogenesis [85]; it also prevents accumulation of TDP-43 in a cell model of ALS through TFEB activation [86].

Although TFEB activates not only lysosomes, but the entire EALP, there are measures that can be employed to specifically target the lysosome. For instance, mutations of *GBA* gene are linked to PD. Whereas homozygous mutations lead to Gaucher disease, heterozygosity is a risk factor for PD. GBA interacts with α-synuclein and disrupts functions of lysosomes, including lysosomal recycling. Whether this defect is caused by accumulation of glucosylceramide, the substrate of GBA, in lysosomes, or a deficiency in GBA non-enzymatic functions is unknown. Regardless, treatment of GBA-deficient fibroblasts with imiglucerase, a recombinant human GBA, is effective in normalizing lysosomal functions. Another potential example is progranulin (PGRN). Whereas a haploinsufficiency of PGRN results in frontotemporal lobar degeneration accompanied by TDP-43 accumulation, a homozygous mutation in PGRN is associated with lysosomal storage diseases, including neuronal ceroid lipofuscinosis and Gaucher disease [87, 88]. Although it remains unknown why a PGRN deficiency induces neurodegenerative diseases, several lines of evidence implicate PRGN in lysosomal function. First, PGRN facilitates the acidification of lysosomes and maturation of cathepsin D (CTSD) [89]. Second, PGRN may act through its C-terminal granulin E domain to function as a chaperone that regulates multiple lysosomal enzymes, including GBA and CTSD [90]. PGRN also has links to TFEB. The promoter of *GRN* contains TFEB binding sites, and PGRN expression is upregulated by TFEB overexpression [14]. Furthermore, a reduction or complete deletion of PGRN changes the expression of genes associated with lysosomal function and lipid metabolism, indicative of lysosomal dysfunction [91].

One of the key determinants of lysosomal function is the luminal pH. For optimal activity of lysosomal enzymes, the lysosomal pH should be 4.5–5.5, largely reflecting the requirements for V-ATPase function. In diverse cell models of proteinopathic neurodegenerative diseases, it is found that lysosomal pH is shifted in the alkaline direction, a change that may be brought about by downregulating the amount and/or H^+^-pumping activity of V-ATPase. The end result of lysosomal alkalinization is decreased fusion between autophagosomes/endosomes and lysosomes, and suboptimal enzyme activities. Regardless of the specific cause, re-acidification of lysosomes tends to normalize fusion as well as degradation functions. Hence, measures that help re-acidify lysosomes may prove useful in ameliorating the progression of proteinopathic neurodegenerative diseases. A recent study reported that acidic nanoparticles may be useful for this purpose [92, 93].

## Possible therapeutics that target normalization of lysosomal acidity: cAMP and Zn^2+^

**Fig. 1:**
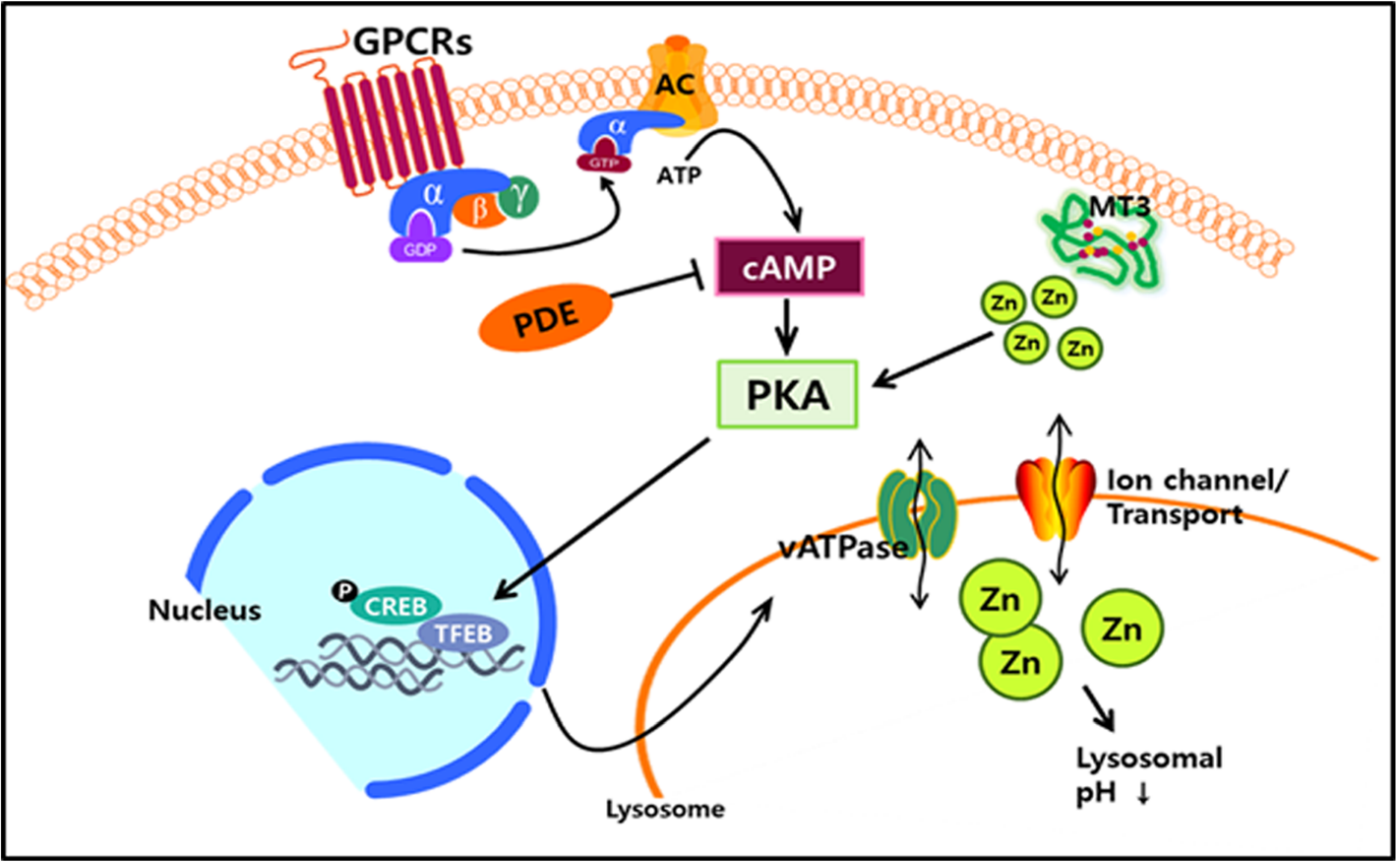
Schematic depiction of cAMP- and Zn^2+^-mediated lysosomal acidification. Increases in cAMP levels through activation of adenylyl cyclase and/or inhibition of PDEs activate PKA. PKA activation leads to increases in lysosomal free Zn^2+^ levels by an as yet unidentified mechanism. Increases in lysosomal Zn^2+^ levels restore lysosomal acidity through an unknown mechanism, even in the presence of BA, a potent and selective inhibitor of the main proton pump, V-ATPase

As potential therapeutics that might help maintain the lysosomal acidity that is critical for lysosomal enzyme activity as well as cargo-lysosome fusion, we would propose to focus on modulating cAMP and Zn^2+^ levels in cells (Fig. 1). For example, it has been shown that raising the level of cAMP re-acidifies lysosomes in mutant PS-1–transfected fibroblasts [31]. Conversely, inhibiting or knocking out adenylyl cyclase, which produces cAMP, results in lysosomal alkalinization [94]. Although the precise mechanism by which cAMP re-acidifies lysosomes has not yet been determined, one study suggested that cAMP may be necessary for the assembly of V-ATPase complex, the main H^+^ pump in the lysosomal membrane [94].

Pharmacologically, there are two ways to increase cAMP levels: activation of adenylyl cyclases, the cAMP-synthesizing enzymes, and inhibition of phosphodiesterases (PDEs), the cAMP-degrading enzymes. Although several drugs indirectly activate adenylyl cyclase by modulating G-protein-coupled receptors, colforsin, a water-soluble form of forskolin used to treat asthma, is the only direct activator in clinical use. In cell models, both forskolin and colforsin have been demonstrated to acidify lysosomes [84].

**Fig. 2:**
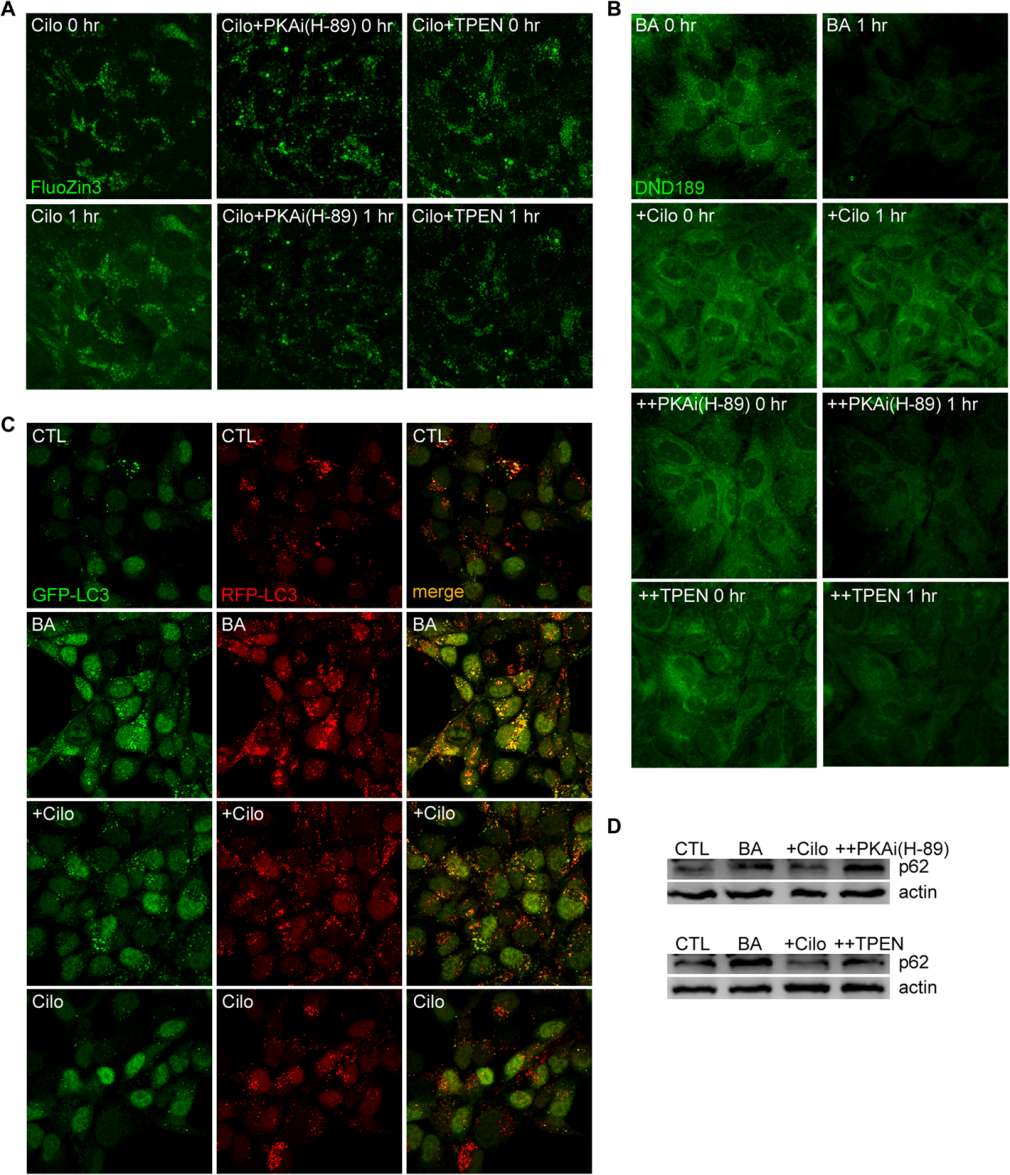
Cilostazol (PDE3 inhibitor) increases lysosomal free Zn^2+^ levels, re-acidifies lysosomes, and promotes autophagy flux. **a**. Fluorescence photomicrographs of FluoZin3-loaded, cultured astrocytes before (left) and after a 1-h treatment (right) with 10 μM cilostazol alone or cilostazol plus the PKA inhibitor H-89 (10 μM) or Zn^2+^ chelator TPEN (500 nM). Cilostazol treatment raised free Zn^2+^ levels in lysosomes, an effect that was blocked by H-89 or TPEN. **b**. Fluorescence photomicrographs of astrocytes loaded with DND189 (a pH-sensitive lysosomal dye) before (left) and after a 1-h treatment (right) with 100 nM bafilomycin A1 (BA) alone, BA plus 10 μM cilostazol, BA plus cilostazol and PKA inhibitor (H-89, 10 μM), or BA plus cilostazol and TPEN (500 nM). **c**. Fluorescence images of H4 cells transfected with both GFP-LC3 and RFP-LC3 obtained after a 6-h treatment with 100 nM BA alone, BA plus 10 μM cilostazol, cilostazol alone, or sham washed (CTL). With BA treatment, GFP fluorescence (left) did not disappear, resulting in many yellow spots in the merged image. Addition of cilostazol substantially reduced GFP signals, resulting in a reduction in yellow spots in the merged image. **d**. Western blots (upper) for p62, a marker of autophagy flux, and corresponding β-actin in samples obtained from astrocytes after a 6-h treatment with 100 nM BA alone, BA plus cilostazol, BA plus PKA inhibitor (H-89, 10 μM), or sham washed (CTL). Another set of Western blots (lower) for p62 and corresponding β-actin in samples obtained from astrocytes after a 6-h treatment with BA alone, BA plus cilostazol, BA plus TPEN, or sham washed (CTL)

The PDE superfamily in humans consists of 12 families whose members are expressed in variable proportions in different cell types. Although all PDE isoforms degrade both cAMP and cGMP, some are more selective for cAMP, and others for cGMP. In contrast to the paucity of adenylyl cyclase activators in human use, a number of PDE inhibitors are in clinical use for various conditions, including erectile dysfunction, asthma and thrombosis prevention, each with well-documented side effects. Hence, repurposing these inhibitors as therapeutics for neurodegenerative diseases might not face large regulatory hurdles. Among PDE inhibitors, cilostazol is known as a PDE3-specific inhibitor that inhibits platelet aggregation, and hence is being used as an antiplatelet agent to prevent coronary or cerebral ischemic events. We experimentally confirmed that cilostazol acidifies lysosomes and increases autophagic flux in astrocytes (Fig. 2). Consistent with this, cilostazol reduces Aβ accumulation in these cells. Recent studies have shown that rolipram, a selective PDE4 inhibitor developed as a potential antidepressant, reduces tau accumulation in a model of tauopathy, likely by activating the proteasome system [95]. However, the authors of this study did not examine the EALP, which might be an additional mechanism of degradation. Although anecdotal evidence such as this exists for the efficacy of PDE inhibitors in various models of neurodegenerative diseases, systematic studies covering the whole spectrum of PDE inhibitors, especially with a focus on their effects on the EALP, have not been conducted, and now seem warranted.

Another potential therapeutic strategy for re-acidification of lysosomes is to raise intracellular or lysosomal free Zn^2+^ levels. Simple exposure of cultured cells to Zn^2+^-enriched media or to a Zn^2+^ ionophore such as clioquinol is sufficient to achieve this effect. Clioquinol increases cytosolic and lysosomal Zn^2+^ levels and activates autophagy, resulting in degradation of mutant huntingtin aggregates [96]. Increasing intracellular or lysosomal Zn^2+^ levels by clioquinol treatment reverse lysosomal pH changes and autophagy arrest (Fig. 2e). Consistent with these changes, clioquinol reduces levels of Aβ or mutant huntingtin in the respective cell models [97]. Also, in preclinical models of neurodegenerative diseases such as AD, clioquinol or its analogue rescues cognitive and behavioral dysfunctions through homeostatic regulation of metal ions such as copper and zinc [98–100].

Lysosomes contain various potential Zn^2+^ transport routes, including ZnT2 (Zn^2+^ transporter-2) and ZnT4, as well as ATP13A2/PARK9. Zn^2+^ transporters (ZnTs) are Zn^2+^-H^+^ antiporters that, upon activation, transfer Zn^2+^ out of the cytosol, thereby reducing cytosolic Zn^2+^ levels [101]. ZnT1 moves Zn^2+^ from the cytosol to the extracellular space, and ZnT2 and ZnT4 transport Zn^2+^ into acidic organelles, such as endosomes, lysosomes, and secretory vesicles. It was recently reported that ZnT2 interacts with V-ATPase, and further that loss of ZnT2 disrupts V-ATPase assembly, impairing vesicle acidification [102]. Another player may be ATP13A2/PARK9, a lysosomal type 5 P-type ATPase. Mutations in ATP13A2 are associated with early-onset Parkinsonism, known as Kufor-Rakeb syndrome (KRS). Studies using *ATP13A2*^−/−^ cells from a KRS patients revealed that *ATP13A2* encodes a Zn^2+^ transporter that serves to sequester Zn^2+^ in endosomes and lysosomes [47]. These studies showed that mutation or knockdown of the corresponding gene results in reduced lysosomal Zn^2+^ levels, increased lysosomal pH and reduced lysosomal degradation, a mechanism that may contribute to the pathogenesis of Parkinsonism. Hence, as is also true in this case, lysosomal Zn^2+^ levels seem to be linked to lysosomal acidification. In theory, the action of ZnTs as Zn^2+^/H^+^ antiporters is predicted to alkalinize lysosomes. One possible explanation for this apparent paradox is that re-acidification may be a physical property of high Zn^2+^ levels in lysosomes, reflecting the fact that Zn^2+^ in solution lowers the pH [103]. Another possibility is that cytosolic or lysosomal Zn^2+^ somehow activates V-ATPase or other indirect routes of H^+^ influx. For instance, Zn^2+^ activates membrane protein kinase C (PKC), which is known to upregulate V-ATPase activity [104]. Although further studies are required to elucidate the underlying mechanism, methods that raise lysosomal Zn^2+^ levels may be helpful for overcoming the lysosomal dysfunction that contributes to the pathogenesis of diverse neurodegenerative diseases. Intriguingly, raising the level of cAMP by cilostazol also results in an increase in lysosomal free Zn^2+^, and chelation of Zn^2+^ with TPEN and PKA inhibitor blocks cAMP effects on lysosomal acidification (Fig. 2). Hence, there may be a mechanistic link between cAMP/PKA (cAMP dependent protein kinase), lysosomal Zn^2+^, and lysosomal pH. How cAMP or PKA mediates increases in lysosomal free Zn^2+^ levels will require future investigation. One caveat in using Zn^2+^ ionophores as therapeutics is the potential toxicity of such agents. Clioquinol was formerly used as an antimicrobial drug in Japan, but was withdrawn because of a serious side effect termed subacute myelo-optic neuropathy (SMON) [105]. In culture conditions, clioquinol can kill neurons and astrocytes by excessively increasing intracellular free Zn^2+^ levels [96]. Also, treatment with clioquinol, likely acting as zinc chelator, has been reported to negatively affect short- and long-term memory and reduce levels of BDNF, synaptic plasticity-related proteins and dendritic spine density [106]. In light of this, developing Zn^2+^ ionophores with optimal physical parameters that do not raise intracellular free Zn^2+^ levels above the cytotoxic threshold may prove to be critical for the clinical use of such agents.

A related finding is that metallothionein-3 (MT3), a CNS-enriched isoform of Zn^2+^-binding metallothionein, has multimodal effects on the EALP in astrocytes. First, Zn^2+^-bound MT3 (Zn-MT3) plays a key role in clathrin-mediated endocytosis [107]. Hence, reduced levels of MT3 may result in aberrant uptake of membrane proteins, such as APP and exogenous proteins secreted from nearby cells. Second, Zn-MT3 contributes to proper maintenance of lysosomal pH in the acidic range. Finally, as a consequence, reduced levels of Zn-MT3 cause arrested autophagy. All these actions appear to be somehow associated with actin polymerization, since 1) MT3 binds to β-actin, and the absence of MT3 inhibits actin polymerization [108]; and 2) inhibitors of actin polymerization replicate the above effects observed in MT3-null astrocytes [107]. Whether these effects of MT3 are mediated by Zn^2+^ released from MT3 or by direct interactions of Zn-MT3 with other proteins, such as actin, remains to be determined. Since MT3 is downregulated in AD brains [109], measures to restore MT3 levels may help normalize the EALP in AD.

In this review, we discussed the possibility that abnormalities in the EALP, especially lysosomal dysfunction and the resultant arrested autophagy, may act as core pathogenic events in diverse proteinopathic neurodegenerative disorders. In addition, we discussed some possible measures that can be taken to normalize lysosomal functions under these conditions, and thereby restore normal flux through the EALP. In particular, we presented evidence showing that measures that raise cAMP and Zn^2+^ levels, as well as those that normalize Zn-MT3 functions, may be effective in restoring lysosomal acidity and catabolic flux through the EALP. Therapeutic strategies for controlling metal dyshomeostasis have been attempted. The use of metal-protein-attenuating compounds (MPACs) such as clioquinol and PBT2 showed the complex actions that are at the same time beneficial or detrimental. Chelation of zinc solubilizes amyloid plaques, but also attenuates synaptic transmission by sequestration of zinc at the synaptic cleft [110–112]. Here, we presented the possibility that an increase of intracellular zinc by raising the level of cAMP or administrating zinc ionophore may be the therapeutics for neurodegenerative diseases by enhancing lysosomal function and consequently decreasing the accumulation of protein aggregates.

## Abbreviations

AD: Alzheimer’s disease
ALS: Amyotrophic lateral sclerosis
APP: Amyloid precursor protein
ATP13A2: ATPase cation transporting 13A2, also known as PARK9
Aβ: Amyloid beta
BACE1: Beta secretase-1
CNS: Central nervous system
CTSD: Cathepsin D
EALP: Endosome-autophagosome-lysosome pathway
ER: Endoplasmic reticulum
GAK: Cyclin G-associated kinase
*GBA*: Glucosylceramidase
HD: Huntington’s disease
KRS: Kufor-Rakeb syndrome
LAMP: Lysosomal-associated membrane protein: ALR: autophagic lysosome reformation
LCL: Neuronal ceroid lipofuscinosis
LIMP-2: Lysosomal integral membrane protein-2
LSDs: Lysosomal storage disorders
M6P: Mannose-6-phosphate
MLIV: Mucolipidosis type IV
MPRs: M6P receptors
MT3: Metallothionein-3
mTOR: Mammalian/mechanistic target of rapamycin
mTORC1: Mammalian/mechanistic target of rapamycin complex 1;ULK: Unc-51-like autophagy activating kinase
MVBs: Multivesicular bodies
NHE: Na^+^/H^+^ exchanger
NPC: Niemann-Pick type C
PD: Parkinson’s disease
PDEs: Phosphodiesterases
PIKFYVE: Phosphoinositide 5-kinase
PKC: Protein kinase C
PSEN1: Presenilin-1
ROS: Reactive oxygen species
SMON: Subacute myelo-optic neuropathy
SOD-1: Superoxide dismutase-1
TFEB: Transcription factor EB
TGN: Trans-Golgi network
TPCs: Two-pore channels
TRPML1–3: Transient receptor potential mucolipin channels 1–3
V-ATPase: Vacuolar ATPase
ZKSCAN3: Zinc finger with KRAB and SCAN domains 3
Zn-MT3: Zn^2+^-bound MT3
ZnT: Zn^2+^ transporter
